# Time-restricted eating, the clock ticking behind the scenes

**DOI:** 10.3389/fphar.2024.1428601

**Published:** 2024-08-08

**Authors:** Aleix Ribas-Latre, Sonia Fernández-Veledo, Joan Vendrell

**Affiliations:** ^1^ Institut d’Investigació Sanitària Pere Virgili (IISPV), Hospital Universitari de Tarragona, Tarragona, Spain; ^2^ CIBER de Diabetes y Enfermedades Metabólicas Asociadas (CIBERDEM)-Instituto de Salud Carlos III (ISCIII), Madrid, Spain; ^3^ Departament de Medicina i Cirugia, Universitat Rovira i Virgili (URV), Tarragona, Spain

**Keywords:** obesity, intermittent fasting, time-restricted eating, chronotype, circadian rhythms

## Abstract

**Introduction:**

Maintaining metabolic balance relies on accumulating nutrients during feeding periods and their subsequent release during fasting. In obesity and metabolic disorders, strategies aimed at reducing food intake while simulating fasting have garnered significant attention for weight loss. Caloric restriction (CR) diets and intermittent fasting (IF) interventions have emerged as effective approaches to improving cardiometabolic health. Although the comparative metabolic benefits of CR *versus* IF remain inconclusive, this review focuses on various forms of IF, particularly time-restricted eating (TRE).

**Methods:**

This study employs a narrative review methodology, systematically collecting, synthesizing, and interpreting the existing literature on TRE and its metabolic effects. A comprehensive and unbiased search of relevant databases was conducted to identify pertinent studies, including pre-clinical animal studies and clinical trials in humans. Keywords such as “Obesity,” “Intermittent Fasting,” “Time-restricted eating,” “Chronotype,” and “Circadian rhythms” guided the search. The selected studies were critically appraised based on predefined inclusion and exclusion criteria, allowing for a thorough exploration and synthesis of current knowledge.

**Results:**

This article synthesizes pre-clinical and clinical studies on TRE and its metabolic effects, providing a comprehensive overview of the current knowledge and identifying gaps for future research. It explores the metabolic outcomes of recent clinical trials employing different TRE protocols in individuals with overweight, obesity, or type II diabetes, emphasizing the significance of individual chronotype, which is often overlooked in practice. In contrast to human studies, animal models underscore the role of the circadian clock in mitigating metabolic disturbances induced by obesity through time-restricted feeding (TRF) interventions. Consequently, we examine pre-clinical evidence supporting the interplay between the circadian clock and TRF interventions. Additionally, we provide insights into the role of the microbiota, which TRE can modulate and its influence on circadian rhythms.

## 1 Introduction

One of the key underlying elements of metabolic homeostasis is a proper transition between periods of nutrient availability and times of scarcity. In mammals, this balance is maintained by accumulating nutrients during feeding periods and releasing stored nutrients during fasting. Main metabolic tissues, including the liver, adipose, and muscle, play well-established roles in energy intake and utilization (Chadt and Al-Hasani, 2020). Furthermore, the gastrointestinal tract, which is initially impacted by ingested nutrients and, in turn, houses its system (the intestinal microbiome), receives special consideration likewise as an active actor in promoting metabolic balance by regulating nutrient metabolism, immune function, and the production of signaling metabolites (Cox et al., 2022). Disrupted homeostasis is shared in many metabolic diseases that generally converge in patients with obesity, diabetes, dyslipidemia, arterial hypertension, and cardiovascular disease. Obesity and its comorbidities have been progressively arising in the last century until reaching epidemic proportions (Saklayen, 2018). A multifactorial and subject-specific process encompassing several associated pathologies without a definitive cure makes the curve difficult to flatten (Guerra et al., 2021). Under this pessimistic paradigm, effective treatment and prevention are the desired weapons for many researchers and the hope for many current and future patients. The diet itself, in terms of quantity, quality, and, more recently, timing, can take part in both treatment and prevention. In recent years, more and more groups have been transitioning from investigating caloric restriction (CR) diets, characterized by a daily controlled reduction in calorie consumption, to the so-called intermittent fasting (IF) diets without a forced caloric restriction (except for the fasting hours/days). Both approaches provide comparable metabolic benefits. However, time-restricted eating (TRE), a specific type of intermittent fasting (IF), garners increased attention due to its practicality and potential to synchronize more effectively with the participant’s natural sleep–wake cycle. Despite pre-clinical studies demonstrating favorable outcomes, no research has aligned participants’ circadian rhythms with a specific TRE protocol.

Human circadian rhythms are influenced by I) the light/dark cycle, also referred to as the solar clock; II) the social clock, which encompasses all social inputs that entrain human sleep/wake cycles; and III) the biological clock, which is partially determined by genetics and explains individual preferences in the timing of sleep and wake, known as chronotypes (Roenneberg et al., 2003). Chronotypes are primarily assessed through questionnaires that categorize individuals based on their tendencies toward extreme, moderate, or slight levels of ‘morningness,’ ‘eveningness,’ or ‘intermediate’ (Horne and Ostberg, 1976; Roenneberg et al., 2003). However, more robust assessments, such as actigraphy, are necessary to validate these subjective categorizations.

Thus, the main focus of this review will weigh heavily on some of the most recent studies looking at TRE and its different types, as well as the basic knowledge behind steaming from both pre-clinical and clinical studies, which contemplate their biological clock as a critical factor to boost the TRE effectiveness. Furthermore, we will briefly discuss the existing relationships among the microbiota, circadian clock, and TRE, highlighting the microbiota as a factor that can be entrained by both TRE and the circadian clock and *vice versa*.

## 2 Nutrient restriction as a therapeutic intervention in obesity

**FIGURE 1:**
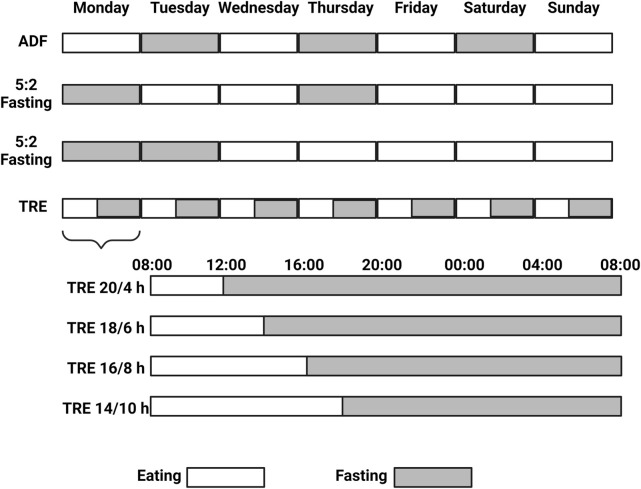
Alternate Day Fasting (ADF), the 5:2 weekly fasting regimen, and daily Time Restricted Eating (TRE) are the most commonly utilized and studied Intermittent Fasting (IF) approaches. 5:2 fasting can be practiced with two consecutive fasting days (or not) across the week, while TRE provides more flexibility and the daily amount (and timing) of the fasting hours can be chosen. Created with BioRender.com.

Nutrient restriction in the form of CR or IF can improve cardiometabolic outputs by reducing body weight (and fat), levels of LDL cholesterol, blood pressure, inflammation, and oxidative stress, in turn, improving the lean mass index and insulin sensitivity (i.e., lower HOMA-IR index) (James et al., 2024). Although CR aims for a sustainable reduction in caloric intake, IF encompasses a broad range of nutrient-restricted strategies. Among the IF diets, alternate-day fasting (ADF), the 5:2 weekly fasting regimen, and daily TRE are the most commonly utilized and studied approaches (Di Francesco et al., 2018) (Figure 1). The fasting state induced by these interventions induces alterations in lipid metabolism by stimulating lipolysis, increasing fatty acid oxidation, enhancing ketogenesis, suppressing lipogenic enzyme activity, and modifying lipid transport, thereby promoting the utilization of stored fats for energy. ADF is the most restrictive option where feasting and fasting days alternate, evoking a stronger feeling of hunger and, thus, a lower adherence (Trepanowski et al., 2017). In addition, 5:2 fasting can help on that avenue, where out of the 7 days of the week, two freely chosen days are reserved for fasting. Whether the fasting days have to be consecutive is not clear in terms of metabolic benefits, leaving the participant the freedom to choose to reach the maximum level of adherence, which, for this strategy, is indeed inferior to a CR diet (Pannen et al., 2021). For example, a study comparing consecutive 36 vs. 60 fasting hours per week within a 4-week intervention in overweight and obese adults showed a modest influence on the gut microbiome and the plasma metabolome, translating into no clinical differences between the groups (Mohr et al., 2022). However, adding one more week of intervention (5 weeks) showed more significant reductions in body weight and waist circumference with 2 consecutive days of fasting compared to 1 day in overweight adults (Arciero et al., 2022). Yet, understanding the systemic adaptions in humans subjected to extreme caloric restriction is very limited. A recent study employing a 7-day water-only fast has shed light on that. Aside from a remarkable weight loss, such an intervention induced systemic proteome reprogramming, which was already evident from the third day and well-conserved across the 12 participants enrolled in the study, with a signature strongly enriched beyond metabolic adaptions (Pietzner et al., 2024). Finally, TRE becomes the least restrictive option, where restriction of food is time-wise and determined across the day, comprising a wide range of restricted-eating windows (from 4 to 10 h) generally across the human active phase (8 a.m.–8 p.m. in average) (Soliman, 2022).

Interestingly, results from two recent meta-analyses comprising over 50 randomized controlled trials comparing the effect of CR and IF interventions for a median period of 2–3 months revealed similar effects in subjects with overweight and obesity in terms of body weight (and composition), plasmatic lipid profile, or insulin resistance improvements (Gu et al., 2022; Ezzati et al., 2023). Extending the period intervention over a year neither showed differences in body weight (Ostendorf et al., 2022) nor even in healthy weight subjects, and the lack of apparent differences was consistent except for a slight reduction in fat-free mass and waist circumference based on a recent meta-analysis composed by 28 trials (Schroor et al., 2024). Only minimal improvements (solely supported by few studies) for IF, when compared to CR, were observed in subjects with overweight and obesity, including a reduction in waist circumference (Gu et al., 2022) and body fat, an improvement in insulin sensitivity (Ezzati et al., 2023), as well as a decrease in inflammatory cytokines accompanied by a reduction in HOMA-IR (Castela et al., 2022). Broader differences have been reported only when both regimens differ in the daily protein intake. IF, accompanied by a 35% daily protein intake compared to CR with a 15% daily protein intake, resulted in reductions in desire to eat, body weight, and total and visceral fat mass accompanied by increments in fat-free mass percent (Arciero et al., 2023). Therefore, both diet interventions are beneficial when compared to a non-intervened group. Still, the preference for one or the other cannot be currently established based on metabolic outputs due to a lack of apparent differences. It has been postulated that the benefits observed under IF regimens are due to a reduction in energy intake, which is the main feature of a CR diet (Liu et al., 2022; Thomas et al., 2022). Thus, a combination of both interventions, subjected to the patient itself, could be the better option. However, evidence in this matter is relatively scarce. Three recent randomized trials in participants with obesity rigorously evaluated the comparative effects of a CR regimen and an identical CR protocol within the framework of TRE interventions, delineated by periods of 8 h/day (Liu et al., 2022), 10 h/day (Thomas et al., 2022), or 12 h/day (Pureza et al., 2020; de; Oliveira Maranhão Pureza et al., 2021). Notably, these investigations revealed no significant disparities in weight loss outcomes. Yet, Oliveira Maranhão Pureza *et al.* found a reduction in body fat composition and waist circumference in obese women (Pureza et al., 2020; de; Oliveira Maranhão Pureza et al., 2021).

Similarly, by doing 4-h eTRE (between 8 a.m. and 12 p.m.) followed by a 20-h fasting period on three non-consecutive days per week and *ad libitum* eating on other days (known as iTRE, which would be an intermediate paradigm between ADF and 5:2 IF) over 6 months, with an additional 12-month follow-up, glucose tolerance was improved compared with CR at month 6, concomitant to a reduction in fasting triglycerides (Teong et al., 2023). Nonetheless, the differences in glucose tolerance were lost at month 18, and no differences were observed in most of the variables studied (i.e., body weight, fat mass, waist circumference, cholesterol levels, etc.) (Teong et al., 2023). Whether another eating window following the participant’s chronotype can display different outcomes has not been addressed. Therefore, given that IF options are “at least” able to mimic CR diets in terms of their impact on metabolic health, the study and further exploitation of TRE, in particular, become more promising due to its greater feasibility and acceptance (Parr et al., 2020a; Kesztyüs et al., 2021) (including in adolescence population (Vidmar et al., 2021)), despite being less successful lowering body weight and mass index when compared to an ADF intervention (Chair et al., 2022). Recent studies have pointed to TRE as a preventive, in addition to an interventional tool for subjects with overweight and obesity (Kesztyüs et al., 2019; Schuppelius et al., 2021; Xie et al., 2022a), opening a dilemma on whether this intervention should rather be seen as a lifestyle implementation.

## 3 Time-restricted eating (TRE), a new strategy of IF

**FIGURE 2:**
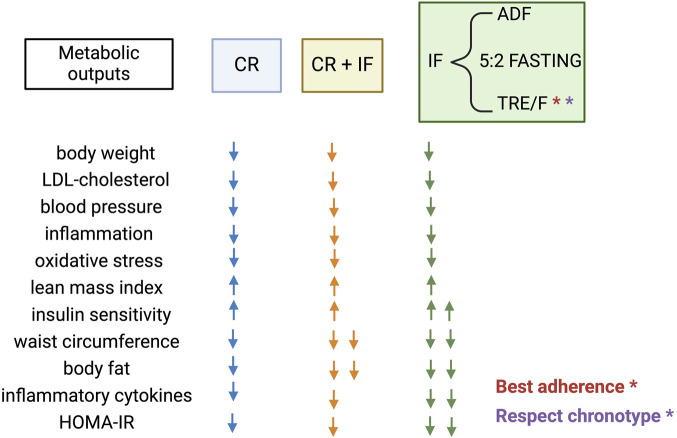
Metabolic outputs produced by Caloric Restriction (CR), different types of Intermittent Fasting (IF) strategies including Alternate Day Fasting (ADF), the 5:2 weekly fasting regimen, and daily Time Restricted Eating (TRE); or the combination of both CR + IF described across non-comparative clinical trials. Created with BioRender.com.

Although no trials directly comparing the effects of ADF, 5:2 fasting, and TRE together have still been performed, TRE possesses two intrinsic advantages compared to the others: i) better adherence due to the lack of forced CR and ii) closer to achieving personalized nutrition according to the inter-subject’s chronotype (Figure 2) (Roenneberg et al., 2003). Yet, at the practical level, TRE intervention is often prescribed without considering the subject’s chronotype, which could mask more beneficial effects. For example, in the “Nurses’ Health Study 2” following 64,615 from 2005 to 2011, evening preference among day workers was associated with a higher T2D risk (Vetter et al., 2015). It has been recently shown that individuals with obesity and a late chronotype present a significantly higher BMI, waist circumference, and hip circumference associated with non-alcoholic steatohepatitis index impairment when compared to individuals with obesity and an early chronotype (Vetrani et al., 2022). A lower adherence to a healthy diet associated with late chronotypes could explain the metabolic impairment observed in this population (Patterson et al., 2016; Bazzani et al., 2022). Yet, a key question remains. How could an early or delayed TRE affect morningness or eveningness chronotypes? Comparing early, late, and self-selected TRE strategies would be necessary to determine whether self-selecting the eating window, which could align with an individual’s chronotype, provides additional benefits for cardiometabolic health in adults with overweight/obesity/T2DM (OOT). The number of clinical trials in single-armed pilot trials, randomized crossover studies, or parallel-arm randomized clinical trials using different forms of TRE in adults with OOT is still not very high. Still, it has dramatically increased since 2018 (Table 1). Nonetheless, those are still in a preliminary phase, and information about diet composition, caloric intake, and other variables, including the inter-subject’s chronotype, is often not contemplated. Furthermore, emerging evidence suggests that sex differences in the effectiveness of these nutritional approaches exist and need to be further addressed (Rius-Bonet et al., 2024).

**TABLE 1:** Recent clinical trials using different forms of Time Restricted Eating (TRE) in adults with overweight, obesity and type 2 diabetes.

Type of intervention	Period of TRE	Type of TRE	Participant	Group	Calorie intake assessment	Chronotype assessment	Major result	References
Single-armed pilot trials	4 weeks	Self-selected (8 h/day)	Nine elder overweight	Baseline transition to TRE (majority 10–18 h) (intermediate)	NO	NO	Body weight ↓ + improvement in quality of life	PMID: 31262054 2019 Anton SD
							14 miRNAs ≠ expressed pre- and post-TRE related to cell growth and survival	PMID: 35565812 2022 Saini SK
Single-armed pilot trials	4 weeks	Intermediate (9 h/day between 10 and 19 h)	19 adults with T2D	Baseline (2 weeks) + TRE (intermediate)	NO	YES	No differences in glycemic control, and physiological and psychological outcomes	PMID: 33105701 2020 Parr EB
Single-armed pilot trials	8 weeks	Self-selected (10 h/day but dinner not later than 19:30)	15 obese men	Baseline (≥12 h/day eating for 2 weeks) + TRE (intermediate)	NO	NO	Stress hormone ↓ + 24 h alteration profile of plasma insulin, NEFA, TG, and GIP levels and SAT transcriptome	PMID: 35912794 2022 Zhao L
							Fasting glucose, glycated hemoglobin, body fat, and weight ↓	PMID: 35150947 2022 Zhao L
Single-armed pilot trials	12 weeks	Self-selected (10 h/day)	19 adults medicated with metabolic syndrome	Baseline for 2 weeks (eating >14 h/day) + TRE	NO	NO	Body weight, waist circumference, % body and visceral fat, blood pressure, atherogenic lipids, and glycated hemoglobin ↓	PMID: 31813824 2020 Wilkinson MJ
Single-armed pilot trials	12 weeks	Self-selected but leaning toward early or intermediate TRE (10 h/day)	16 adults with overweight and obesity	Baseline for 2 weeks (to identify an eating pattern of >14 h/day) + TRE	NO	YES	Body weight, waist circumference, and blood pressure ↓	PMID: 34201442 2021 Prasad M
Single-armed pilot trials	12 weeks	Self-selected (10 h/day)	39 adults with overweight and obesity	Baseline transition to TRE	NO	NO	5% ↓ in body weight for a 26% of the sample + no effect in the blood pressure or lipid profile	PMID: 33508009 2021 Przulj D
Randomized crossover studies	4 days	eTRE (6 h/day between 7:00/9:00 and 13:00/15:00 h) with 3–5 weeks of washout in between	11 adults with overweight	eTRE vs. control (12 h/day between 7:00/9:00 and 19:00/21:00)	YES	NO	Mean ghrelin levels and the 24-h non-protein respiratory quotient ↓	PMID: 31339000 2019 Ravussin E
							↓ 24-h glucose ↑ serum cholesterol and ketones + morning mRNA SIRT1 and LC3A+ altered cortisol and clock gene levels	PMID: 31151228 2019 Jamshed H
Randomized crossover studies	5 days	Intermediate TRE (8 h/day between 10:00 and 18:00 h) with 10 days of washout in between	11 men with overweight and obesity	TRE vs. control (15 h/day between 07:00 and 22:00 h)	YES	YES	Nocturnal glucose AUC ↓	PMID: 32079327 2020 Parr EB
							Rhythmicity of amino acid transporter genes and metabolites	PMID: 32938935 2020 Lundell LS
Randomized crossover studies	7 days	eTRE (9 h/day between 8:00 and 17:00 h) or dTRE (9 h/day between 12:00 and 21:00 h)	15 men at risk for T2D in a	Baseline for 7 days + eTRE vs. dTRE with 2 weeks of washout in between	NO	NO	Compared to baseline, both TRE strategies improve glucose tolerance and decrease fasting TG, with no differences between TRE interventions	PMID: 31002478 2019 Hutchison AT
Randomized crossover studies	9 days	eTRE (6 h/day between 8:00 and 14:00) with 2–3 weeks of washout in between	54 adults with a component of the metabolic syndrome + an eating pattern ≥14 h	eTRE vs. control (≥12 h/day of TRE between 8:00 and 20:00)	YES	NO	24 h mean glucose ↓ and fasted metabolism improvement in response to a mixed model	PMID: 38232698 2024 Dawson MA
Randomized crossover studies	3 weeks	eTRE (10 h/day between 8:00 and 18:00 h) with ≥4 weeks of washout in between	14 adults with T2D	eTRE vs. control (>14 h eating)	NO	YES	24 h glucose homeostasis improvement not accompanied by changes in insulin sensitivity or hepatic glycogen	PMID: 35871650 2022 Andriessen C
Randomized crossover studies	5 weeks	eTRE (6 h/day between 7:00/9:00 and 13:00 h/15:00) with 7 weeks of washout in between	Eight men with overweight, obesity, and pre-diabetes	eTRE vs. control (12 h/day between 7:00/9:00 and 19:00/21:00)	YES	NO	Insulin sensitivity, β-cell responsiveness, blood pressure, oxidative stress improvement, and evening appetite ↓	PMID: 29754952 2018 Sutton EF
Parallel-arm randomized clinical trials	7 weeks	Self-selected (10 h/day but recommended until 20:00 h)	66 obese women and overweight	TRE (n = 33) vs. control (n = 33)	YES	NO	TRE reduces nocturnal glucose	PMID: 36198292 2022 Haganes
Parallel-arm randomized clinical trials	8 weeks	dTRE (8 h/day between 12:00 and 20:00)	21 adults overweight and obese	dTRE (n = 11) vs. control (n = 10)	YES	NO	Reduction in fat mass	PMID: 34042299 2021 Kotarsky CJ
Parallel-arm randomized clinical trials	8 weeks	eTRE (10 h/day between 09:00 and 19:00) or eTRE (12 h/day between 07:00 and 19:00)	78 obese adults	eTRE (n = 39) vs. eTRE (n = 39)	YES	NO	Body weight ↓ in both groups compared to baseline, being TRE (10h/day) more effective (i.e., reduction in basal glucose)	PMID: 33446635 2021 Peeke PM
Parallel-arm randomized clinical trials	8 weeks	dTRE (4 h/day between 15:00 and 19:00) or dTRE (6 h/day between 13:00 and 19:00) with 2 previous baseline weeks	49 obese adults	dTRE (4 h/day) (n = 16) vs. dTRE (6 h/day) (n = 19) vs. Control (n = 14)	NO	NO	Body weight, fat mass, fasting insulin, and oxidative stress ↓ with no differences between groups	PMID: 32673591 2020 Cienfuegos S
Parallel-arm randomized clinical trials	12 weeks	Self-selected (8 h/day)	20 adults overweight	TRE (n = 11) vs. control (eating pattern >14 h/day) (n = 9)	NO	NO	Body weight, lean mass, visceral fat, and the number of eating occasions ↓	PMID: 32270927 2020 Chow LS
							Insulin sensitivity, insulin secretion, or β-cell function ≠	PMID: 36518093 Chow LS (2020)
							Modest quality of life improvement	PMID: 33922683 2021 Crose A
							Bone mineral content ↑	PMID: 33807284 2021 Lobene AJ
Parallel-arm randomized clinical trials	81 days	Self-selected (12 h/day)	58 obese women	Caloric restriction diet (CRD) (n = 27) vs. CRD + TRE (n = 31)	YES	NO	Body fat and waist circumference ↓	PMID: 32428840 2020 Pureza IROM
Parallel-arm randomized clinical trials	12 weeks	eTRE (8 h/day between 08:00 and 16:00) or dTRE (8 h/day between 12:00 and 20:00)	169 adults with metabolic syndrome	TRE (n = 44) vs. low carbohydrate diet (n = 47 vs. combination (n = 44)	NO	NO	↓ Blood pressure, fat mass, and body weight, while improvement in dyslipidemia and glycemic control	PMID: 36220069 2022 He M
Parallel-arm randomized clinical trials	12 weeks	eTRE (10 h/day between 08:00 and 18:00) with 2-week previous baseline	104 adults overweight with T2D	eTRE (n = 54) vs. control (n = 50)	NO	NO	Improvement in HbA1c, fasting glucose, HOMA-β, HOMA-IR, cardiovascular risk lipid markers, body weight, sleep, and quality of life	PMID: 34620199 2021 Che T
Parallel-arm randomized clinical trials	12 weeks	Intermediate (8 h/day between 10:00 and 18:00) with 2-week previous baseline	23 obese adults	iTRE (n = 11) vs. control (n = 10)	NO	NO	↓ Body weight and blood pressure with no changes in body composition and metabolic risk factors	PMID: 29951594 2018 Gabel K
Parallel-arm randomized clinical trials	12 weeks	dTRE (8 h/day between 12:00 and 20:00)	116 adults overweight and obese	dTRE (n = 59) vs. control (n = 57)	NO	NO	No changes in body weight and any other relevant metabolic markers	PMID: 32986097 2021 Lowe DA
Parallel-arm randomized clinical trials	12 weeks	dTRE (8 h/day between 12:00 and 20:00)	32 obese women	dTRE (n = 12) vs. control (n = 20)	NO	NO	Body weight and fat, waist circumference, and CVD risk ↓	PMID: 33407612 2021 Schroder JD
Parallel-arm randomized clinical trials	14 weeks	eTRE (8 h/day between 07:00 and 15:00)	90 obese adults	CRD + eTRE (n = 45) vs. CRD + TRE self-selected >12 h/day; n = 45)	YES	NO	↓ Body weight and fat, blood pressure, and fatigue together with improvement in mood	PMID: 35939311 2022 Jamshed H
							Worse sleep (duration and onset)	PMID: 36518092 Steger FL, 2022
							No changes in eating behavior	PMID: 36575143 Steger FL, 2022
Parallel-arm randomized clinical trials	6 months	dTRE (8 h/day between 12:00 and 20:00)	75 obese adults	dTRE (n = 25) vs. caloric restriction (25) vs. control (25)	NO	NO	↓ Body weight with no changes in euglycemic range, blood pressure, and plasma lipid levels compared to controls	PMID: 37889487 2023 Pavlou V
Parallel-arm randomized clinical trials	6 months	Self-selected (12 h/day)	54 adults with an eating pattern >14 h +1 component of the metabolic syndrome	TRE (n = 28) vs. Control (eating pattern >14 h; n = 26)	NO	NO	No changes in body weight	PMID: 33807102 2021 Phillips NE
Parallel-arm randomized clinical trials	10 months	eTRE (10 h/day within 3 h of waking)	85 obese adults	CRD (n = 42) vs. CRD + TRE (n = 43)	YES	NO	No differences in body weight reduction	PMID: 35470974 2022 Thomas EA
Parallel-arm randomized clinical trials	1 year	eTRE (8 h/day between 08:00 and 16:00)	118 obese adults	CRD (n = 70) vs. CRD + TRE (n = 69)	YES	NO	No differences in body weight and fat, BMI, waist circumferences, blood pressure, and metabolic risk factors	PMID: 35443107 2022 Liu D
Parallel-arm randomized clinical trials	1 year	dTRE (8 h/day between 12:00 and 20:00)	90 obese adults	dTRE (n = 30) vs. CRD (25% caloric reduction; n = 30) vs. control (eating >10 h/day; n = 30)	NO	NO	No differences between dTRE and CRD in weight loss	PMID: 37364268 2023 Lin S
							No changes in mood and quality of life	PMID: 37892388 2023 Lin S
Parallel-arm randomized clinical trials	1 year	Self-selected (12 h/day)	58 obese women	CRD (n = 27) vs. CRD + TRE (n = 31)	YES	NO	↓ Body fat/waist circumference, ↑ axillar temperature, and no changes in body weight	PMID: 32713721 2021 Pureza IROM

The caloric intake of the participants is controlled in 16 out of the 40 clinical trials shown in Table 1, and from those, seven are trials that compare a CR diet to the same CR diet but with the addition of the TRE intervention (Pureza et al., 2020; Jamshed et al., 2022; Liu et al., 2022; Thomas et al., 2022; Steger et al., 2023b; 2023a). In turn, chronotype assessment has been ignored in 36 out of the 40 trials (Table 1), and none of the four left trials (Parr et al., 2020b; 2020a; Prasad et al., 2021; Andriessen et al., 2022) have randomized different subjects’ chronotypes to different TRE windows. So far, this type of clinical trial studying the effect of a TRE window based on the participant’s chronotype does not exist. Yet, this is important considering that late sleepers who followed a 2-week early TRE (eTRE) significantly advanced their sleep timing (Blum et al., 2023), highlighting the power of the intervention in changing crucial behavioral characteristics like sleep.

### 3.1 Self-selected TRE

To align more with the self-chronotype (albeit scientific proof is still required), TRE is self-selected by the participant in 15 out of the 40 studies shown in Table 1. Seven of these 15 studies are single-arm, while the remaining are parallel-arm trials. Regardless of the intervention duration (ranging from 4 to 12 weeks) and the daily hours (between 8–10 h) of self-selected TRE intervention, the metabolic outcomes are consistently beneficial. These benefits include reductions in body weight, waist circumference, percentage of body and visceral fat, blood pressure, atherogenic lipids, dyslipidemia, glycated hemoglobin, and fasting glucose, or increases in bone mineral content and the overall quality of life (Anton et al., 2019; Chow et al., 2020; Wilkinson et al., 2020; Crose et al., 2021; Lobene et al., 2021; Prasad et al., 2021; Przulj et al., 2021; Haganes et al., 2022; He et al., 2022; Saini et al., 2022; Zhao et al., 2022; 2023), although no changes were observed in insulin sensitivity, insulin secretion, or β-cell function (Bantle et al., 2023). By extending the self-selected TRE for a period of 6 months, accompanied by an extension of the TRE window up to 12 h, the decrease in body weight was lost when compared to a group following standard dietary advice and a TRE window superior to 14 h per day (Phillips et al., 2021). Equal outputs were observed in a 1-year intervention parallel-arm trial where the effect of a CR diet was compared to the same CR diet plus a self-selected TRE with an eating window of 12 h per day (de Oliveira Maranhão Pureza et al., 2021), suggesting that a TRE window longer than 10 h is not effective anymore despite stretching the period intervention.

The rest of the studies in Table 1 comprise clinical trials using either i) early (starting no later than 9:00 a.m.), ii) delayed (starting after 12:00 p.m.), or iii) “intermediate” TRE paradigms (starting at 10:00 a.m.).

### 3.2 Early TRE

eTRE during 4 days (6 h/day) reduces 24-h glucose levels and glycemic excursions while increasing serum cholesterol, ketones, and mRNA SIRT1 and LC3A in the morning (Jamshed et al., 2019). Similarly, eTRE during 9 days (6 h/day) also lowers mean 24-h glucose and glycemic variability while improving fasted but not postprandial metabolism in response to a mixed meal (Dawson et al., 2024). However, this short-term eTRE does not affect body weight, macronutrient absorption, gastrointestinal transit time or hydrogen production, resting energy expenditure, or the thermic effect of food, plasma metabolome, fecal microbiome, or sleep (Dawson et al., 2024). Extending eTRE for 3 weeks (10 h/day) improves 24-h glucose homeostasis independently of changes in insulin sensitivity or hepatic glycogen in subjects with T2D (Andriessen et al., 2022). However, eTRE for 5 weeks (6 h/day) improves insulin sensitivity, β-cell responsiveness, blood pressure, and oxidative stress (Sutton et al., 2018); those effects are maintained in subjects with diabetes and overweight or participants with metabolic syndrome when the eTRE is extended up to 12 weeks and the TRE window up to 8 (He et al., 2022) or 10 h (Che et al., 2021). Thus, this intervention seems to require some adaptation to maximize beneficial effects. However, it is unclear whether longer fasting hours produce more beneficial metabolic outputs in the obese population. The intervention period generally adjusts the TRE window to maintain adherence. Thus, the longer the intervention, the more the TRE window is stretched. Yet, in front of the same intervention period, more daily fasting hours seem to maximize the metabolic outputs. One parallel-armed trial comparing the effect of an eTRE (12h/day) to another eTRE (10h/day) showed more metabolic improvements for the latter, including significant effectiveness in decreasing basal glucose (Peeke et al., 2021). When eTRE is combined with a CR and such intervention is compared to a CR diet alone, outputs are beneficial when each group is compared to baseline but inexistent when both groups are compared despite long-period interventions (10–12 months) (Thomas et al., 2022). In contrast, a similar parallel-armed trial comparing a CR diet together with an eTRE (8 h/day) to a control group composed of the same CR diet together with a self-selected TRE with a daily eating window superior to 12 h during 14 weeks, showed an improvement in body weight, fat mass, heart rate, insulin resistance, glucose, blood pressure, and mood (Jamshed et al., 2022; Steger et al., 2023a), despite no changes in diet quality, meal frequency, and other eating features when compared to the control group (Steger et al., 2023b). Remarkably, this clinical trial was applied to a population with obesity who usually woke up between 4:00 and 9:00 a.m., suggesting that an eTRE intervention in participants who seem to have an early chronotype based on their waking time (Jamshed et al., 2022; Steger et al., 2023b; 2023a) is more effective.

### 3.3 Intermediate and delayed TRE

Conversely, when the TRE intervention is initiated 1–3 h later (‘intermediate’ paradigm), the effects are negligible after 5 days of intervention (8 h per day, iTRE) (Parr et al., 2020a) or relatively modest after 4 weeks of intervention (9 h per day, iTRE), as indicated by changes in the nocturnal glucose area under the curve (Parr et al., 2020b), based on findings from a crossover and a single-arm study, respectively. Remarkably, in the latter study, Parr et al. examined a population of 11 men who were overweight and obese and who displayed “definite morning” (n = 2), “moderate morning” (n = 3), or “intermediate” (n = 6) chronotypes (Parr et al., 2020b), based on the morning–eveningness questionnaire (Horne and Ostberg, 1976). Since more than half of the participants displayed an intermediate chronotype, performing an iTRE was a good choice despite the fewer participants. Yet, whether subject-specific differences were observed due to the difference in chronotype was not reported, and indeed, it would be difficult to conclude due to the small sample size. Therefore, additional studies in this area are warranted. Nevertheless, extending the intermittent time-restricted eating (iTRE) intervention to 12 weeks (8 h per day) resulted in reductions in both blood pressure and body weight (Gabel et al., 2018). This suggests that a more extended intervention period may yield better outcomes, although further studies are necessary to substantiate this finding and provide more detailed insights.

Finally, three (two-arm) studies employing delayed TRE (dTRE) (starting at noon and for 8 h) for 8 and 12 weeks, respectively, reported mild (Kotarsky et al., 2021) to no metabolic improvements (Lowe et al., 2020) compared to control. Only Schroder J et al. reported reduced body weight, body fat composition, and waist circumference after a 12-week intervention and 8-h dTRE strategy (Schroder et al., 2021). Interestingly, among those three parallel-armed trials, Lowe et al. was the study presenting the larger sample population. Reducing the time-eating window in another three parallel-arm trial studying the effect of 4-h and 6-h dTRE paradigms (starting at 15:00 and 13:00, respectively) during 8 weeks resulted in clinical and metabolic improvements (including body weight, fat mass, fasting insulin, insulin resistance, and oxidative stress) independently of sleep quality (Cienfuegos et al., 2022) when compared to control, albeit not any difference was observed between dTRE groups (Cienfuegos et al., 2020). Similar outputs were found in two dTRE starting at noon and for 8 h but throughout 6 (Lin et al., 2023a) or 12 months (Pavlou et al., 2023). Thus, except for overweight older men and women (age range = 65–74 years) who lost weight under a 6-week dTRE (Domaszewski et al., 2023), dTRE seems to be more effective as more fasting hours are included in the intervention or longer the intervention is. In this sense, long interventions of 6 and 12 months resulted in a decrease in the TRE time window when comparing the final time point to baseline (Lin et al., 2023a; Pavlou et al., 2023), suggesting that the participants at this point are sufficiently adapted to naturally modify their feeding behavior, without altering their mood or quality of life (Lin et al., 2023b). Ramadan is another good example of fasting hour extension and feeding behavior change. Despite contemplating the most night-shift dTRE window studied, Ramadan practice also translates into clinical and metabolic improvements in the population with obesity (not shown in Table 1) (Al-Rawi et al., 2020; Zouhal et al., 2020).

### 3.4 Comparative studies

Thus, despite a wide range of different TRE options, conclusions regarding the best choice are challenging due to a lack of comparative studies. One recent 7-day randomized crossover study with 15 men at risk for T2D and 2 weeks of washout in between compared the effects of a 9-h eTRE (8:00–17:00 h) vs. a 9-h dTRE (12:00–21:00 h) paradigm (Hutchison et al., 2019). Although TRE improved glucose tolerance and fasting glucose and triglycerides compared to baseline, no differences were observed between TRE strategies (Hutchison et al., 2019). Whether non-crossover randomized studies performed in participants with OTT comparing longer interventional time show differences have not been investigated. Whether there were differences in terms of chronotype across the 15 men participating in this study, which could impact the results (Vetter et al., 2015), was also not investigated. A similar study in adults with BMI 19–27 kg/m^2^ and with not extreme morning or evening chronotypes following an 8-week daytime eating schedule (8 a.m.–7 p.m.), compared to a delayed eating schedule (12 p.m.–11 p.m.), revealed weight loss and improvements in energy metabolism and insulin when the TRE was carried out during the earlier schedule (Allison et al., 2021). This would suggest that in a population with an intermediate chronotype, the eTRE would work better. However, the same comparative study in subjects with obesity and the same period of time in terms of intervention has not been addressed. Despite not having any information about the participant’s chronotype, similar outputs were observed in healthy participants without obesity subjected to an 8-h eTRE (between 06:00 and 15:00) when compared in parallel to an “intermediate” TRE (between 11:00 and 20:00) carried out during 5 weeks (Xie et al., 2022b).

On the other hand, trials directly comparing different TRE paradigms in terms of the quantity of restricted-eating hours (independently of timing of eating windows) in adults with OOT are likewise scarce. Four studies respectively compared 6 h (Sutton et al., 2018; Jamshed et al., 2019; Ravussin et al., 2019) or 10 h (Peeke et al., 2021) of restricted eating to a control group with 12 h of restricted eating. In both situations, 6 or 10 h of restricted eating was sufficient to improve metabolic markers, including insulin sensitivity, β-cell responsiveness, blood pressure, oxidative stress (Sutton et al., 2018) or reduce glucose levels (Jamshed et al., 2019), and body weight (Peeke et al., 2021) when compared to the 12 h restricted-eating control group. Yet, further effects cannot be ruled out because most clinical trials use a representative population sample with a habitual eating pattern of more than 12 h a day as a control (Parr et al., 2020b; Chow et al., 2020; Lundell et al., 2020; Wilkinson et al., 2020; Crose et al., 2021; Lobene et al., 2021; Prasad et al., 2021; Zhao et al., 2022; 2023). An average daily period of more than 14 h from the first to the last food consumption event was reported in a cohort of 156 healthy participants monitored for 21 days via a smartphone application (Gill and Panda, 2015). Although this technology is progressively used to enroll participants (Parr et al., 2020a; Chow et al., 2020; Prasad et al., 2021), more observational studies will shed light on country-specific differences (if any).

**FIGURE 3:**
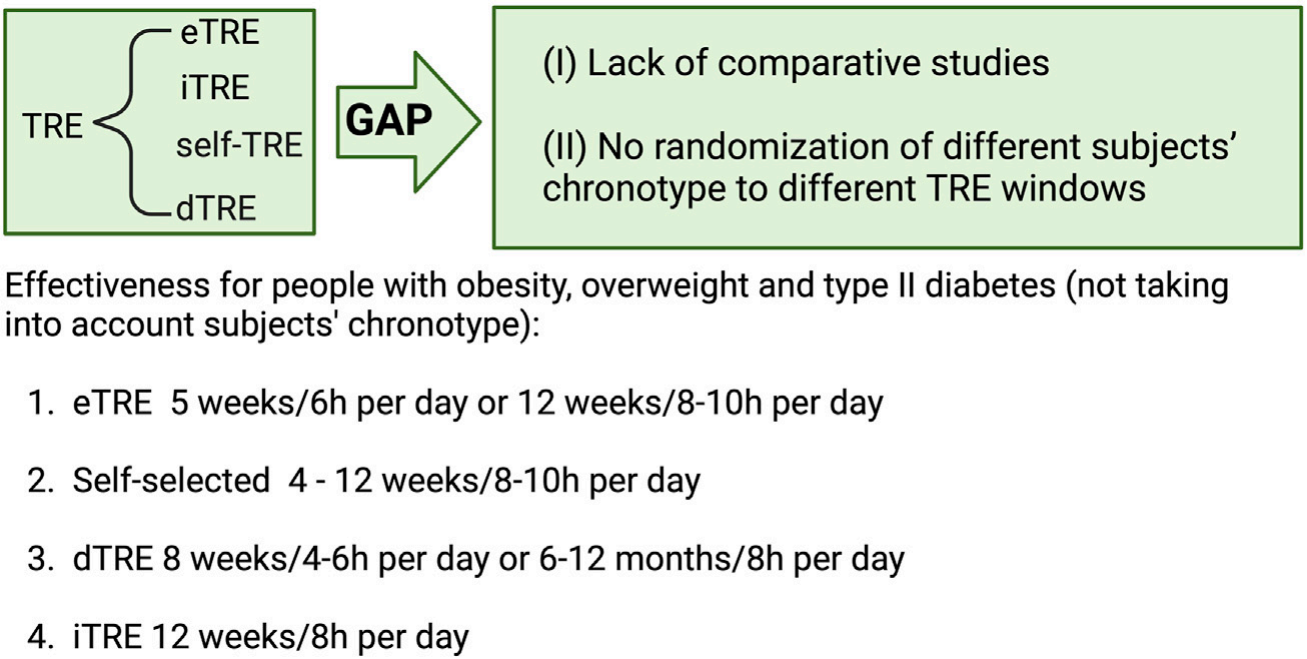
Intervention length and daily fasting hours of different types of Time Restricted Eating (TRE) including early (eTRE), intermediate (iTRE), self-TRE or delayed (dTRE) to ensure effectiveness in subjects with overweight, obesity and type 2 diabetes, according to non-comparative studies in which subject’s chronotype has been not randomized. Created with BioRender.com.

Figure 3 broadly summarizes this section, classifying the various TRE strategies in terms of their effectiveness in adults with overweight, obesity, or type II diabetes (OTT). However, this figure provides only a snapshot of the current knowledge. It should not be applied in clinical settings until more comparative studies are conducted and the chronotype of the participants is determined and aligned with the TRE intervention.

## 4 TRE and the circadian clock

Despite the lack of human trials employing TRE interventions according to the participant’s chronotype, animal models of obesity have shown metabolic improvements when time-restricted feeding (TRF), in this case, is implemented during the animal’s active phase (Chaix et al., 2014; 2019; Chung et al., 2016; de Goede et al., 2019; Smith et al., 2019; Hou et al., 2021). However, whether TRF at the optimal time provides superior health outcomes compared to ADF and 5:2 fasting in mice remains unclear due to a lack of comparative studies. Compared to the control mice on a high-fat diet (HFD) *ad libitum*, ADF, and TRF protocols, they resulted in weight loss, while TRF and 5:2 fasting reduced visceral adiposity (Habiby et al., 2024). In another similar study, utilizing different fat percentages in the HFD, ADF, and TRE interventions consistently led to reduced weight and fat mass gain, lower fasting glucose, insulin, and HOMA-IR, and improved glucose tolerance, particularly in the ADF group (Smith et al., 2019). Except for fasting glucose and glucose tolerance, no additional differences were observed among the IF (and the CR control) groups, suggesting that, similar to humans, there are no apparent differences between CR and IF (and among IF paradigms) in murine models (Smith et al., 2019). Behaviorally, CR impacts food intake patterns more severely than TRF, resulting in a drastic reduction in the duration of daily food intake and a shift in the temporal pattern of wheel running (Acosta-Rodríguez et al., 2017). This suggests that CR associated with IF diet is a major factor behind the metabolic improvements. Other studies in mouse models employing IF in ADF support this notion (Henderson et al., 2021), while other studies indicate an apparent independence (Zheng et al., 2024). Sex- and age-dependent effects of CR in murine models could account for such inconsistencies (Suchacki et al., 2023).

Although the fact of presenting metabolic disturbances have been associated with a disrupted circadian clock in human and animal models, the disruption of the circadian clock, in turn, has been associated with an increased risk of cardiometabolic disorders (Morris et al., 2015; Shimizu et al., 2016). Whether the disruption of the circadian clock is previous to the appearance of the metabolic disorder has not been demonstrated. Yet, animal models of circadian misalignment, including jet lag (Kettner et al., 2015) or genetic ablation of some specific clock gene (Turek et al., 2005; Paschos et al., 2012), clearly promote the beginning of the pathologic process. The circadian clock comprises peripheral clocks (spread throughout the body) and a central clock in the hypothalamic suprachiasmatic nucleus (SCN).

Given that every cell in the body possesses its clock, the latter functions as a cell-specific temporal sensor capable of organizing many aspects of the cellular metabolism in front of extracellular inputs. Although photic signals from the light–dark cycle mainly entrain the central clock, peripheral clocks can be influenced by other external cues, including diet and fasting/feeding cycles (Green et al., 2008). Thus, communication across the different clocks around the body is crucial in maintaining metabolic balance. Rodent models have suggested that restricted feeding at the wrong time (i.e., rest/light phase) (Damiola et al., 2000) or high-fat diet (HFD) given *ad libitum* can impair such communication (Kohsaka et al., 2007; Eckel-Mahan et al., 2013), while restricting HFD feeding during the rodent active phase for 12 weeks can re-establish this communication accompanied by an improvement in several metabolic markers including the body weight and composition among others (Hatori et al., 2012; Chaix et al., 2014; 2019). Thus, unlike human studies, animal models have shown the involvement of the clock as a mechanism to restore metabolic disturbances induced by obesity after a TRF intervention. In other words, TRF seems to boost the clock while promoting health. In this sense, the role of the clock has to be seen as the controller of the circadian pattern of wakefulness and sleep (i.e., feeding and fasting), while the TRF/E as a “clock agonist” that facilitates the alignment of those diurnal physiological events. TRF intervention can even restore the metabolism of diet-induced obesity mice lacking a clock core gene (via genetically modified mouse models) (Chaix et al., 2019). Although this finding suggested that TRF can restore obesity and metabolic-related disturbances independently of the clock, these different clockless models phenotypically display arrhythmicity in the food intake pattern, and the TRF can re-establish it (Chaix et al., 2019). Mechanistically, TRF exerts an epigenetic control of the pancreatic beta cell function, reverting, in turn, insulin resistance despite the circadian clock disruption (Brown et al., 2021). Thus, the metabolic reprogramming caused by the re-installment of the normal oscillatory food intake pattern, ultimately driven by the TRF, seems to compensate for the absence of a specific clock gene. More specific mechanisms that shed light on the interrelation between TRF/E and the clock system at the molecular level are warranted. Still, so far, insulin sensitization seems to be the strongest link. Several studies restoring insulin function in insulin resistance mouse models have shown a parallel re-setting of the clock (Yamajuku et al., 2012; Eckel-Mahan et al., 2013; Tseng et al., 2015; Ribas-Latre et al., 2019).

## 5 The case for the gut microbiota

Because the link between TRE and the clock system seems to be the oscillatory pattern of food intake, a third variable within the equation strongly affected by food intake can connect both TRE and the clock system. Such a candidate meeting the criteria is the gut microbiota. The gut microbiota comprises a wide range of microorganisms, including bacteria, archaea, fungi, protists, and algae, required to preserve the host’s metabolic balance (Berg et al., 2020). Interestingly, the microbiota’s composition and (thereby) function exhibit daily oscillations intimately related to the oscillatory food intake pattern of the host (Thaiss et al., 2014; Tuganbaev et al., 2020). Not only food timing but also the quality and quantity of food finally translated into dietary components, either metabolized by the host or escaping from the host’s absorptive process (e.g., fiber), modulates the microbiota composition (Zarrinpar et al., 2014; Klingbeil and de La Serre, 2018; Nova et al., 2022). In turn, the microorganisms composing the microbiota generate metabolites or signaling molecules able to positively or negatively impact the host’s physiology, depending on the amount of microbe-related compounds generated (directly related to the microbial diversity, species abundance, or the phylum level) and the host susceptibility to uptake or defend from them (Krautkramer et al., 2021). The list of bioactive microbial metabolites includes (and is not limited to) short-chain fatty acids (e.g., acetate, propionate, butyrate, and colicin), carboxylic acid intermediates (e.g., lactate and succinate), secondary bile acids (e.g., lithocholic acid (LCA), and deoxycholic acid (DCA) and derivatives), pattern receptor recognition (PRR) ligands (e.g., lipopolysaccharide (LPS), peptidoglycan, lipoteichoic acid (LTA), polysaccharide A, bacterial DNA, and secreted microbial proteins), flavonoids (e.g., quercetin, apigenin, and naringenin), lipids and fatty acids (linoleic acid and arachidonic acid), vitamins (group B, K2, A, C, and D), protein–amino acid derivatives (e.g., trimethylamine oxide, taurine, branched-chain amino acids (BCAAs), tryptophan, lysine, and l-glutamine), or other classes including indole-3-propionic acid (I3PA), indole-3-aldehyde (IAId), indoxyl sulfate (IS), or tryptamine (Li et al., 2018; Spivak et al., 2022).

One general trend associated with improved host outcomes is its microbiota’s bacterial diversity (Lozupone et al., 2012). Germ-free mice colonized with microbes isolated from humans or rodents with cardiometabolic disorders, which present lower (and different) bacterial diversity within their microbiota, develop metabolic complications, including weight gain (Turnbaugh et al., 2006; Ridaura et al., 2013; Thaiss et al., 2016a), fatty liver (Murakami et al., 2016), hypertension (Shi et al., 2021), and impaired glycemic response (Suez et al., 2014; Gérard and Vidal, 2019). When inducing weight gain accompanied by adverse metabolic effects and gut microbiota dysbiosis characterized by lower microbial diversity (Liu et al., 2020), HFD feeding disrupts the host’s clock system (Kohsaka et al., 2007; Eckel-Mahan et al., 2013) concomitant with a dampened oscillation of the gut microbiota and gut-derived metabolic factors (Zarrinpar et al., 2014; Martchenko et al., 2020). For example, the circadian secretion of GLP-1 from L-cells is attenuated due to HFD feeding. However, by transferring fecal microbiota from a mouse-eating standard chow into a mouse-eating HFD, GLP-1 circadian secretion is restored (Martchenko et al., 2020). Additional models of circadian desynchrony (and metabolic imbalance), such as jet lag or core clock ablation, similarly display a dampened gut microbiota oscillation (Thaiss et al., 2014; 2016b; Zarrinpar et al., 2014; Heddes et al., 2022). In turn, microbial rhythms’ disruption (or absence) alters several murine tissues’ host rhythmic transcriptome, epigenome, and metabolome (Thaiss et al., 2016b; Kuang et al., 2019; Weger et al., 2019). Therefore, maintaining metabolic homeostasis implies a fine-tuned regulatory checkpoint composed of a dynamic and diverse healthy microbiota and a functional clock system. In this sense, TRE/F not only reinforces peripheral clock rhythmicity but also could expand microbiota diversity and heighten its diurnal oscillation depending on the specific intervention and the host’s microbiota state (Hu et al., 2018; Van Der Merwe et al., 2020; Ye et al., 2020; Ratiner et al., 2022).

Any TRE strategy could lead to different effects, as has been observed in the case of other IF paradigms addressed to people with overweight and obesity. ADF for 1 (ADF-1) or 2 (ADF-2) consecutive days of fasting in a 4-week intervention leads to different outputs. Although ADF-1 increases the abundance of Ruminococcaceae Incertae Sedis and Eubacterium fissicatena families, ADF-2 also increases the abundance of Ruminococcaceae Incertae Sedis and decreases the levels of the *Eubacterium ventriosum group*, translating at the metabolome level with increases in serine, trimethylamine oxide (TMAO), levulinic acid, 3-aminobutyric acid, citrate, isocitrate, and glucuronic acid compared to ADF-1 (Mohr et al., 2022). Along the same line, an every-other-day (ADF) IF regimen in obese mice results in a shift in the gut microbiota composition, leading to elevation of the fermentation products acetate and lactate, which translates into a selective stimulation of beige fat development within white adipose tissue and a consequent amelioration of obesity (Li et al., 2017). Confirmatory results in terms of browning have been recently observed using the same IF paradigm, with additional inhibitory lipid absorptive effects driven by Akkermansia muciniphila (Yang et al., 2023), while a consistent increase in the abundance of health-associated genera, including *Lactobacillus,* and an increase in the abundance of Firmicutes, accompanied by a decrease in the proportion of Bacteroidetes and Verrucomicrobia, have also been observed in a genetic model of obesity, specifically in the db/db mice (Beli et al., 2018).

Alternatively, a 3-week 5:2 fasting intervention in human volunteers with varying BMI levels resulted in a significant enrichment of *Parabacteroides distasonis* and *Bacteroides thetaiotaomicron*, which inversely correlated with parameters related to obesity and cardiovascular diseases (Hu et al., 2023). In contrast, an 8-week intervention in participants with metabolic syndrome increased the relative abundance of Ruminococcaceae at the family level and *Roseburia* at the genus level, belonging to Firmicutes. These alterations were significantly associated with cardiovascular risk factors and led to distinct genetic shifts in carbohydrate metabolism within the gut microbiome (Guo et al., 2021). By conducting a similar intervention in participants with overweight and obesity but with fasting on three non-consecutive days per week, an overall shift in the microbiota structure and diversity from baseline to 3 months was observed, with an increase in *Akkermansia* abundance, which all together correlated with a reduction in the waist circumference (Stanislawski et al., 2021).

In the case of a TRE, participants with obesity enrolled in a 12-week TRE regimen below 12 h/day showed a minimal effect in microbiota diversity and composition compared to a control group with more than 12 h/day eating. In this case, only an increase in the frequency of Lachnospiraceae, Parasutterella, and Romboutsia at the end of the study was observed, with uncertain clinical relevance (Ferrocino et al., 2022). Indeed, a recent systematic review focusing on the clinical effects of TRE in the gut microbiota concluded that only two human studies showed a beneficial correlation between microbiota changes driven by the TRE intervention and the host’s metabolic (HDL cholesterol) or anthropometric parameters (body mass index) (Pieczyńska-Zając et al., 2023). Yet, in this systematic review, only seven clinical trials between 2020 and 2022 were included (three out of the seven were observational studies using Ramadan as a TRE), highlighting the lack of knowledge in this field. Furthermore, several possible confounding factors, study-design limitations, the host’s lifestyle itself, and the microbiota’s great level of flexibility in front of any dietetic regimen, could be behind the weak causal relationship between IF and the improvement of gut microbiota-related outcomes (Paukkonen et al., 2024). Despite similar confounding factors, the impact of TRE in gut microbiota on the lean population seems to be more consistent and in a good direction when a similar TRE protocol is applied. Three studies employing a very late 8-h TRE during 25–26 days, similar to IF-Ramadan, generally revealed an increase in bacterial diversity together with an increase in Prevotellaceae, Bacteroideaceae, and Firmicutes, while pathogenic bacteria were decreased (Zeb et al., 2020a; 2020b; Khan et al., 2022). Similar outputs have been observed in murine models of diet-induced obesity. TRE for 6–8 h during the active phase in obese mice increases α-diversity microbiota (Van Der Merwe et al., 2020) and healthy micro-groups, including Firmicutes phylum, Clostridia class, Ruminococcaceae family, or *Roseburia* genus, while detrimental groups are inhibited (Hu et al., 2018). This is consistent with another study where restricting food consumption during the active phase was accompanied by a CR (Zhang et al., 2019). Interestingly, by switching from active-phase TRF-CR to *ad libitum* feeding, the gut microbiota returned to the state resembling that of mice fed standard chow *ad libitum*, but that of rest-phase TRF-CR mice was still significantly different from the other two groups, reinforcing the importance of the timing of feeding (Zhang et al., 2019).

Thus, the general notion across pre-clinical and clinical studies is that IF regimens (ADF, 5:2 fasting, or TRE) enhance the microbiota structure and diversity, translating into healthy outputs. Yet, the number of studies is still considerably low, and consistency and reproducibility regarding specific microbes being modulated by different IF strategies, particularly TRE interventions, are lacking. Therefore, this field needs to be further exploited, and conclusions regarding the impact of IF on microbiota are far from being reached.

## 6 Discussion

Modern life with constant access to noise, artificial light, rigid schedules, processed food, etc., accompanied by sleep loss, late of eating, or lack of stable eating patterns with a tendency to skip meals (Gill and Panda, 2015), can lead to circadian misalignment, or in other words, chronic “Social Jet Lag” negatively impacting metabolism (Wittmann et al., 2006). An extreme example of that is the case of rotating shift workers, who exhibit an increased risk of developing metabolic disorders (Cheng et al., 2021; Yang et al., 2021; Bayon et al., 2022) and some types of cancer (Härmä et al., 2023). On the other hand, a rhythmic food intake pattern based on the rhythmic pattern of wakefulness and sleep is crucial to sustain the clock system and partially prevent metabolic derangements. One novel tool feasible for rotating shift workers (Manoogian et al., 2022) to promote and increase the robustness of the rhythmic food intake pattern and mitigate metabolic disturbances is TRE. So far, TRE is the only approach that contemplates time daily to maximize health. Aside from the quantity and the quality of food ingested, the timing of food consumption likewise matters. Studies have shown that in front of the same meal in terms of composition and caloric power, a different metabolic response is triggered when the time of eating is different. Studies in healthy adults have shown that evening vs morning eating results in higher postprandial glucose concentrations and GIP (Takahashi et al., 2018) and lower postprandial insulin secretion and GLP-1 responses (Jakubowicz et al., 2015).

Intriguingly, these effects can be mitigated by skipping the evening meal (Tsuchida et al., 2013; Nas et al., 2017) or manipulating the quantity of food administered at night. For example, by transferring 100 kcal of fat intake from night (20:30–05:00) to earlier periods, systemic low-density lipoprotein (LDL) cholesterol levels are reduced (Chen et al., 2019). Having dinner 3 hours earlier (6 p.m. vs. 9 p.m.) improves 24-h blood glucose levels and boosts lipid metabolism after breakfast the next day (Nakamura et al., 2021). Importantly, this effect seems independent of the type of food since the blood glucose levels caused by the consumption of high glycemic index foods are higher when food is offered at night, compared to light hours in healthy subjects (Gibbs et al., 2014). In contrast, shortening the time lapse between one meal and another by having lunch earlier increases glucose tolerance, accompanied by increased carbohydrate oxidation and fasting resting energy expenditure (Bandín et al., 2015). Similar observations have been shown in studies comprising subjects with T2D (Jakubowicz et al., 2015), overweight, and obesity (Garaulet et al., 2013). Mechanistically, a hormonal imbalance driven by evening eating could play a role. For example, the concurrence of high melatonin levels at night and high glucose levels after a late dinner impairs glucose tolerance, specifically in homozygous carriers of the MTNR1B risk allele (Lopez-Minguez et al., 2018), pointing to the melatonin receptor *MTNR1B* as an important factor. Similarly, the lower concentrations of epinephrine/norepinephrine and higher concentrations of acylated ghrelin after the evening dinner (at 20:00) could also explain such unfavorable effects at night since the balance between these hormones was shifted after a morning meal (at 8:00) (Bo et al., 2017).

Thus, eating earlier rather than later is a good strategy to improve metabolic health, which suggests that implementing TRE early in the morning could be a good approach. Based on what is known, TRE should respect breakfast time since skipping this early meal could impair rather than improve metabolic balance (Betts et al., 2014; Ballon et al., 2019). Therefore, better than reducing the number of meals per day, modulating the timing of those while keeping consistency confined in a shorter eating window (Fleischer et al., 2022) could be sufficient to improve self-metabolic responses to feeding. Whether the feasting time window needs to be necessarily earlier than later and which amount of fasting hours are required to obtain better metabolic outcomes need to be personalized, considering the person’s chronotype. A 12-week randomized (including 1-year follow-up) double-blind, parallel-group controlled trial studying the effectiveness of a hypocaloric diet in weight loss in people with overweight and obesity via the comparison of a hypocaloric dietary treatment to the same regimen but adjusted to the chronotype’s participants concluded that the chronotype-adjusted diet significantly reduced body weight, BMI, and waist circumference when compared to the other group (Galindo Muñoz et al., 2020). Indeed, despite the lack of studies randomizing participants in a TRE intervention according to their chronotype, several examples of the chronotype-driven benefits are observed at different levels. For example, executive function in pre-adolescents and academic performance in adolescents is improved when school timing is better aligned with their biological rhythms (students with early chronotypes perform better in the morning, and the opposite is true for students with late chronotypes) (Hahn et al., 2012; Lara et al., 2014; Goldin et al., 2020). In addition to cognitive performance, the chronotype should be considered when scheduling training sessions to promote a faster recovery in athletes. Morning-type young participants subjected to high-intensity interval training in the evening presented impairment in sleep quality accompanied by signs of pre-fatigue and wellness the following day (Vitale et al., 2017; Saidi et al., 2023). Likewise, exercise following the appropriate chronotype can mitigate the physiopathological condition for some illnesses. For instance, performing exercise in synchrony with the chronotype may be essential to decrease migraine load in chronic migraine (Malek et al., 2023), while an improvement in parameters such as HbA_1c_, fasting blood glucose, HDL-LDL cholesterol, triglyceride, total cholesterol, functionality, and quality of life has been observed in individuals with T2D (Menek and Budak, 2022).

Furthermore, chronotherapy, taking into account the patient’s chronotype, could also be applied to treat several pathologies, including inflammatory bowel disease (Swanson et al., 2023), rheumatoid arthritis (To et al., 2011), hypertension, and cardiovascular disease (Festus et al., 2024) or asthma (Pincus et al., 1995) among others. A thousand cycling genes expressed across the body encode proteins that either transport or metabolize drugs or are themselves drug targets, highlighting the importance of considering circadian and behavioral rhythms when studying drug response (Ruben et al., 2018). Further studies will build up from this baseline to better understand the TRE consequences and improve its efficacy.
